# Accuracy in the estimation of children's food portion sizes against a food picture book by parents and early educators

**DOI:** 10.1017/jns.2018.26

**Published:** 2018-12-27

**Authors:** Kaija Nissinen, Liisa Korkalo, Henna Vepsäläinen, Päivi Mäkiranta, Leena Koivusilta, Eva Roos, Maijaliisa Erkkola

**Affiliations:** 1 School of Food and Agriculture, Seinäjoki University of Applied Sciences, PO Box 412, 60101 Seinäjoki, Finland; 2 Department of Food and Nutrition, University of Helsinki, PO Box 66, 00014 University of Helsinki, Finland; 3 Department of Social Research, Faculty of Social Sciences, University of Turku, Assistentinkatu 7, 20014 Turku, Finland; 4 Folkhälsan Research Center, Paasikivenkatu 4, 00250 Helsinki, Finland; 5 Department of Public Health, Clinicum, University of Helsinki, PO Box 20, 00014 University of Helsinki, Finland

**Keywords:** Food photographs, Validation, Visual perception, Early education professionals, Parents, DAGIS, Increased health and wellbeing in preschools

## Abstract

Validated methodological aids for food quantification are needed for the accurate estimation of food consumption. Our objective was to assess the validity of an age-specific food picture book, which contains commonly eaten foods among Finnish children, for parents and early educators in estimating food portion sizes. The food picture book was developed to assist in portion size estimation when filling in food records in the Increased health and wellbeing in preschools (DAGIS) study. All ninety-five food pictures in the book, each containing three or four different portion sizes, were evaluated at real-time sessions. Altogether, seventy-three parents and 107 early educators or early education students participated. Each participant evaluated twenty-three or twenty-four portions by comparing presented pre-weighed food portions against the corresponding picture from the food picture book. Food portions were not consumed by participants. The total proportion of correct estimations varied from 36 % (cottage cheese) to 100 % (fish fingers). Among the food groups, nearly or over 90 % of the estimations were correct for bread, pastries and main courses (‘piece products’ such as meatballs and chicken nuggets). Soups, porridges, salads and grated and fresh vegetables were least correctly estimated (<65 % correct estimations). There were small differences in evaluations of berries and fresh fruits, warm vegetables and pastries between the parents and early educators, but other estimations were mostly similar. The children's food picture book was found to be a useful aid for the estimation of food portion sizes. Parents and early educators evaluated the portion sizes with similar accuracy.

Accurate portion size estimation is challenging but essential for food exposure assessment. Food photographs are a useful aid for the quantification of food items and in increasing the accuracy of portion size estimations^(^^1^^–^^4^^)^, even when used by the children themselves^(^^5^^–^^7^^)^. There are also food pictures with additional photographs of food leftovers^(^^6^^,^^8^^)^, which may further improve the accuracy of portion size estimations. With children of preschool age or under, the leftovers may play an important role in food consumption's estimation. Andersen *et al*.^(^^9^^)^ recommend the use of a structured food record booklet with an adapted food picture book and household measures as a best practice in dietary assessment among preschool-age children. Compared with weighing, food photographs are considered more practical and less burdensome as a portion size estimation aid^(^^10^^)^. The validity of the photographs against the weighing method has been shown to be good^(^^1^^)^. In recent years, food photographs have also been developed to be used in digital form^(^^11^^–^^13^^)^.

Compared with adults, children are more vulnerable to nutritional risks. Consequently, portion size estimations are of special interest when determining the energy and nutrient intake of children. Children's food selection can differ from adults. The content of the food picture book targeting for children must therefore be based on the food culture and selection of foods of the target group^(^^4^^,^^7^^,^^14^^)^. Also, portion sizes need to be age-adjusted^(^^4^^,^^7^^,^^15^^)^. To get a full picture of the diet of young children, we most often need to combine food recordings from multiple surrogates: parents, other guardians, and early educators in a nursery or preschool.

The objective of the present study was to assess the validity of the age-specific food picture book, which contains a selection of commonly used foods among Finnish children, for estimating food portion sizes in general and among parents and early educators separately. To our knowledge, there are no previous studies comparing the portion size estimations between parents and early educators.

## Methods

### Development of the food picture book for the DAGIS study

**Fig. 1.**
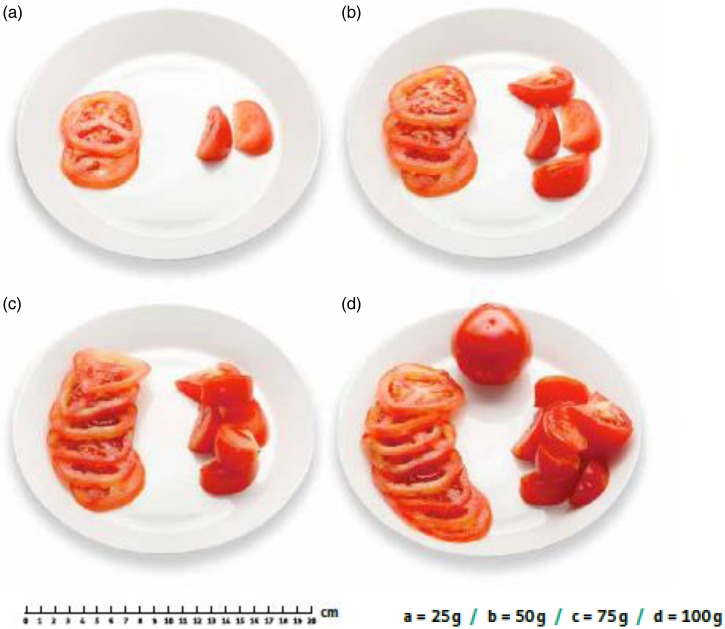
Example of one photograph series of an individual food item (tomato) from the food picture book.

The food picture book was developed for the purposes of the Increased health and wellbeing in preschools (DAGIS) study^(^^16^^)^. Selection of the foods and portion sizes were derived from the LILLA intervention study conducted among preschool-aged children in the Helsinki metropolitan area. The portion sizes were based on the average portion sizes consumed by 3- to 5-year-old Finnish children^(^^17^^)^. Size B was the average portion, size A was half of it, size C was the average × 1·5 and size D was 2 × the average. However, before the final definition of the portion sizes, trial photographs were taken, and the pictures were piloted by two nutritionists active in clinical work and commented on by nutrition researchers to ensure that the differences between portion sizes were practical and visually detectable. General packaging sizes influenced some of the food amounts (e.g. a whole chocolate bar or a small box of raisins). The final selection of food for the picture book contained ninety-five commonly known Finnish foods divided into ten categories: (1) drinks, (2) bread, (3) spreads, cold cuts and cheeses, (4) vegetables, (5) fruits and berries, (6) porridges and cereal, (7) potatoes, pasta, rice and mixed vegetables, (8) main courses, (9) snacks, desserts, and pastries and (10) confectioneries and snacks. There were mainly four portion sizes presented per food (Fig. 1).

The food photographs were taken by a professional photographer with a Canon EOS 5d Mark II camera and EF24-70mm f/2.8L USM objective. The photographs were taken at a 45° angle except for fruits, which were photographed vertically. Lighting was executed with an Elinchrom 400bx soft box. The size of the picture files was 20 cm × 5·16 cm and had a resolution of 300. All the pictures were embedded on a white surface, and the shadows were made with photograph editing software.

### Ethical review

The study was conducted according to the guidelines laid down in the Declaration of Helsinki. The study obtained a favourable ethical statement from the University of Helsinki Review Board in Humanities and Social and Behavioural Sciences in June 2016.

### Study design

In our study, we used the visual perception method^(^^18^^)^, which means that participants were asked to make direct comparisons between food shown on a plate and food in a photograph. We pre-tested the validity study protocol at Seinäjoki University of Applied Sciences in April 2015.

Portion size estimations were carried out in real-time sessions. Seven separate validation sessions were organised: five sessions in preschools for parents and early educators and two sessions at Seinäjoki University of Applied Sciences where students of early childhood education participated in the study. The portion sizes on the plates were the same as in the food picture book. An Excell SI-130 electronic scale with accuracy of 2 g was used for weighing. We used the same recipes, and the food was laid out just like it was in the picture book. The plates, cups, glasses and bowls were different from those in the photographs. The portions and the order of the presentation of the food items were randomly allocated between the study tables (Excel RAND function).

In each evaluation session, we had four tables with the same food items but different portion sizes. The participants were guided to one of the tables in order of arrival, and they evaluated the portion sizes of all food items presented on the evaluation table. The trained assistants supervised the sessions and gave oral instructions to the participants. The assistants were trained by the first author (K. N.). Every table had its own assistant in case the participants had questions. The participants were asked to compare the presented food portions with the food portions shown in the food picture book and to choose the portion size that they thought best demonstrated the portion on the plate. There was no option to choose a portion size between the ones shown in the food picture book. The estimation took on average 20–30 min. In addition to the evaluation form, participants filled in a short background questionnaire including questions about their age, sex, height, weight, number and age of children, education and information about whether the participant was a guardian, early educator or student of early childhood education.

Trained assistants checked the filled evaluation forms and questionnaires. If there were missing answers, the participant was kindly asked to complete the evaluation form or questionnaire if he/she thought it was possible.

In these seven sessions, all ninety-five food items and all portion sizes (i.e. every individual picture) in the food picture book were estimated by participants.

### Participants

Our aim was to recruit two groups of similar sizes: parents and early educators. First, we asked from the head of early education in Seinäjoki the list of all the preschools with a kitchen very close to a proper dining room with enough space to organise evaluation sessions. We then invited all these five preschools to participate in the study, and they all consented. After that we invited early educators and parents of children in these preschools to participate. Information letters with consent forms were distributed to the parents of children at the preschool and a short oral presentation of the study and information letters with a consent form were given to the staff in every participating preschool. The inclusion criteria for early educators were that they regularly take part in the children's daily meals. The number of early educators was too small to achieve a sufficient sample size. Therefore, we also invited all final-year students of early childhood education at Seinäjoki University of Applied Sciences and Vocational Education Center Sedu, Kauhajoki who had recently had their training period at the preschool. An oral presentation about the study was given and information letters with a consent form were distributed to these students.

### Justification of sample size

The target sample size of estimates per food item was calculated through the formula as described in Noether^(^^19^^)^ (significance level 0·05, power 80 %):

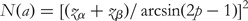


According to these calculations, fifty-four estimates per food item were needed.

### Statistical analyses

Every picture in a series was given a number (1 = the smallest amount) in running order to the largest amount. The difference between the picture chosen by the participant and the actual picture was calculated, and the results are presented as percentages of participants choosing the correct picture (difference 0) or distant picture (difference ± 1 or more). The differences between the weight of the food item in the chosen picture and the weight of the food item on the table (g) were calculated (estimated – actual), and 95 % CI are presented. The geometric mean of the ratio of estimated to actual portion size is presented with 95 % CI for every individual food item. Further analyses were done after combining the original ninety-five foods into twenty-three food groups (e.g. bread, soups, drinks) in order to interpret the results of similar foods in compact categories. The proportions (%) of correct estimations by food group and in parents and early educators are separately presented. The geometric mean of the ratio of estimated to actual portion size in g by food group and by parents and early educators separately were also calculated and presented with 95 % CI. After calculating the ratio of estimated portion size (g) divided by actual portion size (g) and taking the natural logarithm of this ratio, independent-samples *t* tests were conducted to test the differences of evaluations between the parents and early educators. Bland–Altman plots with 95 % limits of agreement were made for the visual inspection of agreement between the actual and estimated portion sizes. Analysed food groups were chosen according to food groups of interest in the DAGIS study (fruits, berries and vegetables, sugar-containing foods) as well as those food groups of which estimation turned out to be challenging. To give a better display of many overlapping observations, the jitter.normal function was used to add random variation in Bland–Altman plots.

Statistical analyses were carried out using IBM SPSS Statistics version 24 and R (version 3.1).

## Results

Altogether, 3798 evaluations were made by 180 participants. The evaluations per food item varied from fifteen to fifty-seven because we had different numbers of participants depending on the size and participation rate in different preschools. On average, forty-one evaluations per food item were made.

The background characteristics of the participants are presented in Table 1. Most of the participants were women (83 %) and had secondary-school or lower-level education (57 %). The mean age was 33 (sd 10) years. Early educators were mainly women and were younger and less educated than parents.

**Table 1.** Characteristics of the participants (Numbers and percentages; mean values and standard deviations)

	All participants		Parents*		Early educators†		
	*n*	%	*n*	%	*n*	%	*P*
Participants	180	100	73	41	107	59	
Sex							<0·001‡
Women	150	83	49	67	101	94	
Men	30	17	24	33	6	6	
Age (years)							0·002§
Mean	33·0		35·7		31·2		
sd	10·4		7·2		11·8		
BMI (kg/m^2^)							0·719§
Mean	24·6		24·5		24·7		
sd	3·8		3·8		3·8		
Level of education							<0·001‡
Secondary school or lower	102	57	28	38	74	69	
Bachelor's degree	60	33	30	41	30	28	
Master's degree or higher	18	10	15	21	3	3	

* Includes three guardians other than parents.

† Including fifty-four early educators and fifty-three final-year students of early childhood education.

‡ *χ*^2^ Test between parents and early educators.

§ Independent-samples *t* tests between parents and early educators.

### Percentages of correct, adjacent or distant picture, and differences measured in g

The percentages of participants choosing the correct picture (difference 0) or distant picture (difference ± 1 or more) from the picture series for individual food items are shown in Table 2. The proportion of correct estimations for individual food items ranged from 100 % (fish fingers, Karelian pastries) to 36 % (cottage cheese). The percentage of participants selecting distant (±1) pictures varied from 0 to 50. Differences between the actual and estimated portion size in g for individual food items varied from −40·5 (95 % CI −58·9, −22·2) g for cottage pie to 49·1 (95 % CI 49·1, 65·8) g for vegetable soup on a deep plate (see Table 2). In general, according to the geometric mean, portion sizes of food items were more often underestimated (forty-seven out of ninety-five) than overestimated (twenty-one out of ninety-five food items), but as can be seen in Table 2, the difference between real and estimated weight was very small in most cases.

**Table 2.** Participants (*n* 180) choosing the correct (0), −1, +1 or more distant portion number compared with the actual portion number, difference between the actual and estimated portion size in g and the ratio of the estimated to the actual portion size in g (Percentages, mean values, geometric means and 95 % confidence intervals)

	No. of estimations	Difference between estimated portion number compared with actual portion number (% participants choosing)					Difference* (g)		Ratio†	
Food/ingredient		0	−1	1	<−1	>1	Mean	95 % CI	Geometric mean	95 % CI
Fish fingers	52	100	0	0	0	0	0	–	1·06	1·00, 1·00
Karelian pastries	26	100	0	0	0	0	0	–	1·00	1·00, 1·00
Rye bread	57	98	0	2	0	0	0	−2·5, 2·5	1·00	1·00, 1·00
Minced-meat beef	37	97	0	0	3	0	−2·4	−7·4, 2·5	0·96	0·93, 1·00
Chicken nuggets	37	97	0	0	3	0	−1·1	−3·3, 1·1	1·00	0·95, 1·00
Bread cheese	49	96	0	4	0	0	0·8	−0·3, 2·0	1·01	1·00, 1·03
Mango mash on plate	24	96	0	4	0	0	0·4	−0·4, 1·3	1·04	1·01, 1·08
Egg	27	96	4	0	0	0	−0·6	−1·7, 0·6	0·96	0·94, 0·99
Cauliflower	57	95	5	0	0	0	−1·6	−3·6, 0·4	0·96	0·94, 0·99
Spinach crêpe	37	95	3	0	3	0	−3·2	−8·1, 1·6	0·94	0·91, 0·99
Sausage	37	95	0	3	3	0	−1·4	−5·4, 2·7	0·99	0·96, 1·04
Meatballs	37	95	5	0	0	0	−1·6	−3·9, 0·7	0·97	0·96, 0·99
Cinnamon bun	38	95	0	5	0	0	1·8	−0·8, 4·4	1·05	1·02, 1·09
Smoothie	36	94	3	3	0	0	0	−4·0, 4·0	1·00	0·99, 1·01
Chicken, strips	53	94	2	4	0	0	1·5	−6·6, 9·5	0·97	1·03, 1·20
Chicken fillet	53	94	2	4	0	0	0·5	−1·2, 2·1	1·01	1·00, 1·03
Chocolate	53	94	3	2	0	0	−1·3	−3·8, 1·2	0·96	0·93, 1·00
Cookies	53	93	8	0	0	0	−0·2	−0·4, 0	0·96	0·95, 0·98
Apple, slice	26	92	8	0	0	0	−2·3	−5·8, 1·2	0·94	0·92, 0·97
Weetabix	32	91	9	0	0	0	−0·9	−2·0, 0·1	0·97	0·96, 0·99
White bread	57	90	4	2	4	0	−1·1	−2·6, 0·4	0·97	0·95, 0·99
Ham	36	89	3	3	0	6	0·2	−0·1, 0·4	1·02	1·01, 1·03
Sweets/candy	53	89	9	2	0	0	−4·5	−8·9, −0·2	0·96	0·94, 0·99
Carrots, cooked	57	88	9	4	0	0	−2·9	−6·1, 0·3	0·96	0·93, 1·00
Ice cream	53	87	13	0	0	0	−4·2	−7·3, −1·0	0·92	0·89, 0·95
Baguette	50	86	10	4	0	0	−1·0	−3·0, 1·0	0·95	0·92, 1·00
Croutons	36	86	8	6	0	0	−0·3	−1·6, 1·0	0·98	0·97, 1·01
Hamburger	28	86	14	0	0	0	−4·3	−8·4, −0·1	0·96	0·95, 0·98
Strawberries	32	84	16	0	0	0	−7·8	−14·5, −1·2	0·93	0·91, 0·95
Hot chocolate	36	83	5	11	0	0	2·8	−4·2, 9·7	1·02	1·00, 1·04
Pear	22	82	0	14	0	5	16·8	−1·7, 35·3	1·08	1·06, 1·12
Crispy rice cereal	32	81	6	13	0	0	0·6	−0·9, 2·2	1·04	0·98, 1·12
Commercial baby food	37	81	8	8	3	0	−5·7	−17·9, 6·5	1·01	0·95, 1·08
Raisins	53	81	15	2	2	0	−3·4	−5·8, −1·0	0·93	0·90, 0·96
Rye loaf, slice	15	80	20	0	0	0	−5·0	–	0·74	0·72, 0·77
Apple pie	39	80	18	0	3	0	−4·8	−7·6, −1·3	0·91	0·89, 0·94
Apple	24	79	4	17	0	0	12·9	−8·4, 34·2	1·06	1·03, 1·10
Chocolate cereals	32	78	13	9	0	0	−0·3	−2·0, 1·4	1·00	0·97, 1·03
Potatoes, mashed	32	78	13	6	0	3	0	−10·2, 10·2	1·00	0·98, 1·04
French fries	32	78	9	13	0	0	−0·8	−7·8, 6·2	1·02	0·98, 1·07
Spaghetti, cooked	37	78	11	8	3	0	−4·1	−12·3, 4·2	0·95	0·91, 1·00
Pizza, mini round	37	78	16	5	0	0	−3·2	−7·8, 1·3	0·96	0·93, 0·99
Pizza, slice	37	78	0	16	5	0	0·8	−6·5, 8·1	1·02	0·97, 1·07
Citrus fruits	26	77	0	23	0	0	27·7	6·1, 49·3	1·17	1·12, 1·23
Brown gravy	39	77	18	5	0	0	−5·1	−10·4, 0·2	0·91	0·87, 0·96
Crisps	53	77	21	2	0	0	−2·2	−3·6, −0·8	0·87	0·84, 0·92
Water	49	76	16	4	4	0	− 6·1	−12·4, 0·2	0·93	0·91, 0·97
Lasagne	37	76	16	8	0	0	−12·2	−27·6, 3·3	0·96	0·93, 1·01
Sausage–egg–potato hash	37	76	14	8	3	0	−5·4	−14·9, 4·0	0·94	0·91, 0·98
Risotto	37	76	24	0	0	0	−12·2	−19·4, −4·9	0·89	0·87, 0·92
Vegetable soup in bowl	53	76	2	23	0	0	20·7	8·2, 33·3	1·08	1·05, 1·12
Rice porridge	32	75	6	20	0	0	8·8	−4·2, 21·7	1·06	1·03, 1·11
Salad	57	74	7	16	0	4	1·8	−1·0, 4·7	1·10	1·05, 1·16
Feta cheese	53	74	25	0	2	0	−2·8	−4·2, −1·5	0·89	0·87, 0·92
Nuts	53	74	25	0	2	0	−2·8	−4·2, −1·5	0·90	0·88, 0·93
Beefy macaroni casserole	37	73	18	8	0	0	−8·1	−24·1, 7·9	0·92	0·88, 0·97
Ketchup	41	73	22	2	2	0	−1·8	−3·1, −0·6	0·84	0·80, 0·89
Juice	36	72	28	0	0	0	−11·1	−17·6, − 4·6	0·90	0·88, 0·93
Kiwi fruit	32	72	13	10	6	0	−3·1	−8·0, 1·8	0·94	0·90, 0·98
Flat rye bread (with hole)	42	71	14	14	0	0	0	–	1·00	0·96, 1·05
Margarine	49	69	0	29	0	2	1·6	0·9, 2·4	1·14	1·11, 1·19
Pineapple	32	69	25	0	6	0	−8·9	−14·3, −3·5	0·83	0·80, 0·87
Cream cheese	50	68	16	16	0	0	0	−0·8, 0·8	1·02	0·98, 1·08
Mixed vegetables, cooked	37	68	22	8	3	0	−7·6	−14·0, −1·2	0·92	0·88, 0·96
Frankfurter gravy	37	68	19	14	0	0	−2·7	−12·3, 6·9	0·98	0·95, 1·02
Banana	24	67	25	8	0	0	−6·7	−16·2, 2·9	0·95	0·94, 0·98
Macaroni, cooked	37	65	3	32	0	0	9·8	2·8, 16·8	1·14	1·10, 1·18
Rice, cooked	37	65	24	3	8	0	−9·5	−15·1, 3·9	0·85	0·82, 0·89
Minced-meat sauce	37	65	0	32	3	0	13·5	3·4, 23·6	1·17	1·13, 1·23
Berry quark	53	64	26	4	6	0	−13·6	−20·7, −6·4	0·85	0·82, 0·90
Banana, pieces	24	63	29	8	0	0	−0·4	−3·8, 3·0	0·87	0·84, 0·92
Grapes	32	63	13	25	0	0	6·3	−4·7, 17·2	1·07	1·04, 1·11
Mango mash in bowl	32	63	22	6	9	0	−10·3	−18·4, −2·3	0·90	0·87, 0·94
Pea–maize–carrot mix	37	62	35	3	0	0	15·8	10·7–20·9	1·41	1·35, 1·48
Cottage pie	37	62	35	0	3	0	−40·5	−58·9, −22·2	0·84	0·82, 0·88
Liver sausage	36	61	6	33	0	0	1·4	0·4, 2·3	1·14	1·10, 1·19
Raspberry jam	41	61	30	7	2	0	−2·2	−3·7, −0·7	0·83	0·78, 0·89
Berry mix	32	60	41	0	0	0	−15·6	−23·1, −8·1	0·76	0·73, 0·81
Ground beef soup in bowl	53	60	34	4	2	0	−27·4	−41·0, −13·7	0·84	0·80, 0·89
Ground beef soup on deep plate	53	60	23	13	2	2	−6·8	−21·8, 8·2	0·97	0·93, 1·02
Cucumber	57	58	30	0	12	0	−9·6	−13·3, −6·0	0·73	0·70, 0·78
Sweet bell pepper	57	58	42	0	0	0	−4·9	−6·7, −3·1	0·74	0·71, 0·79
Yoghurt	53	57	26	9	6	2	−12·3	−23·0, −1·5	0·91	0·87, 0·97
Raspberry soup	53	57	17	8	19	0	−18·9	−28·7, −9·0	0·82	0·78, 0·88
Tomato	57	56	25	0	19	0	−15·8	−21·1, −10·5	0·78	0·75, 0·82
Watermelon	32	56	19	25	0	0	2·5	−7·1, 12·1	1·02	0·98, 1·07
Potatoes, cooked	32	56	13	31	0	0	4·7	−4·5, 13·8	1·14	1·08, 1·21
Cheese	50	54	12	32	0	2	0·9	0·5, 1·7	1·10	1·05, 1·15
Salmon	37	54	19	27	0	0	3·1	−5·0, 11·3	1·10	1·01, 1·12
Carrots, raw	57	51	25	4	21	0	−16·6	−23·3, −9·9	0·67	0·63, 0·73
Four-grain porridge	32	50	3	38	0	9	38·8	19·4, 58·0	1·24	1·20, 1·31
Carrots, grated	57	49	46	4	2	0	−8·4	−11·7, −5·1	0·77	0·74, 0·82
Vegetable soup on deep plate	53	45	4	50	0	2	49·1	49·1, 65·8	1·22	1·18, 1·28
Muesli	32	44	50	0	6	0	−6·6	−9·3, −3·7	0·65	0·62, 0·70
Cottage cheese	53	36	36	0	28	0	−9·2	−11·5, −7·0	0·62	0·59, 0·66

* Estimated – actual.

† Ratio = estimated/actual in g.

**Fig. 2.**
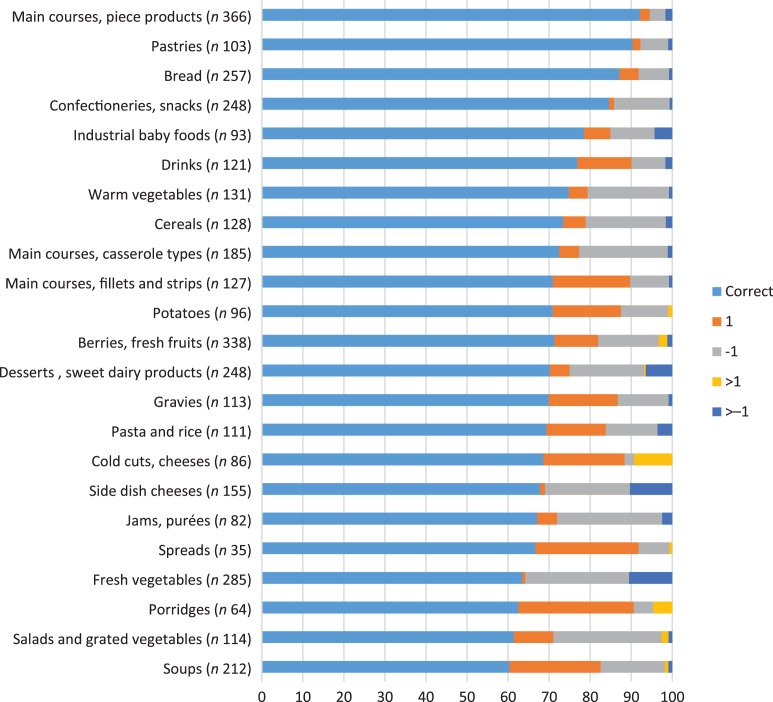
Percentages of participants choosing the correct, adjacent (+1/−1) or distant (>+1/−1) portion number compared with the actual portion number (*n*, number of estimations/food group).

Among all food groups, over 60 % of estimations were correct. The correct estimation was achieved in over 90 % of estimations of the main courses that are served as ‘pieces’ (e.g. meatballs, chicken nuggets, fish fingers) and pastries (Fig. 2). Also, bread and confectioneries and snacks were estimated correctly in over 80 % of the cases. The largest percentage difference between the estimated and correct pictures was found for soups: 60 % of the estimations were correct (Fig. 2). Additionally, less than 65 % of the estimations were correct in the cases of salads and grated vegetables, porridges and fresh vegetables. Fresh vegetables were far more often underestimated than overestimated.

### Comparisons of portion size estimations between the parents and the early educators

In general, estimations of parents and early educators were similar. Early educators’ estimations ranged on average from the underestimation of fresh vegetables by 20 % to the overestimation of porridges by 18 % (Table 3). Parents’ estimations ranged on average from the underestimation of jams and purées by 24 % to the overestimation of porridges by 14 %. Among parents, fresh vegetables were underestimated by 22 %. Statistically significant differences between parents and early educators were found in the cases of warm vegetables, berries, fresh fruits and pastries (Table 3).

**Table 3.** Ratios of estimated to actual portion size (g) by food group (total), by parents (*n* 73) and early educators (*n* 107) (Geometric means and 95 % confidence intervals)

	Total (*n* 180)		Parents* (*n* 73)		Early educators† (*n* 107)		
Food group	Geometric mean	95 % CI	Geometric mean	95 % CI	Geometric mean	95 % CI	Difference between parents and early educators: *P*‡
Fresh vegetables	0·79	0·77, 0·82	0·78	0·74, 0·83	0·80	0·77, 0·84	0·799
Salads and grated vegetables	0·91	0·89, 0·94	0·93	0·90, 0·97	0·91	0·87, 0·95	0·712
Warm vegetables	1·00	0·97, 1·04	1·06	1·02, 1·12	0·96	0·92, 1·01	0·039
Berries–fresh fruits	1·00	0·99, 1·02	0·96	0·95, 0·98	1·04	1·03, 1·07	0·018
Drinks	0·95	0·94, 0·97	0·96	0·93, 0·98	0·95	0·93, 0·98	0·779
Desserts, snacks	0·94	0·91, 0·97	0·96	0·91, 1·01	0·93	0·90, 0·96	0·480
Confectionery, snacks	0·93	0·92, 0·95	0·94	0·92, 0·97	0·92	0·91, 0·95	0·672
Jams, purées	0·85	0·81, 0·90	0·76	0·70, 0·84	0·88	0·84, 0·94	0·200
Bread	1·02	1·01, 1·05	0·99	1·02, 1·06	1·04	1·02, 1·06	0·164
Pastries	0·95	0·94, 0·98	0·89	0·86, 0·93	0·98	0·96, 1·01	0·034
Spreads	1·06	1·04, 1·10	1·02	1·05, 1·12	1·08	1·05, 1·12	0·338
Cold cuts, cheese	1·13	1·09, 1·17	1·06	1·09, 1·22	1·15	1·09, 1·22	0·169
Side dish cheeses	0·86	0·83, 0·89	0·85	0·82, 0·89	0·86	0·82, 0·90	0·815
Porridges	1·16	1·13, 1·20	1·14	1·09, 1·20	1·18	1·14, 1·23	0·661
Cereal	0·91	0·89, 0·93	0·89	0·87, 0·92	0·93	0·91, 0·97	0·300
Potato	1·00	0·98, 1·03	0·96	0·93, 1·00	1·04	1·01, 1·08	0·161
Pasta, rice	0·99	0·96, 1·02	0·99	0·96, 1·03	0·98	0·94, 1·04	0·954
Main courses, casserole type	0·90	0·88, 0·93	0·89	0·86, 0·93	0·92	0·89, 0·97	0·552
Main courses, piece products	0·98	0·97, 1·00	0·99	0·99, 1·00	0·98	0·95, 1·01	0·595
Main courses, fillets and strips	1·01	0·98, 1·05	1·02	0·98, 1·08	1·00	0·96, 1·05	0·553
Gravies	0·99	0·95, 1·04	0·99	0·94, 1·07	0·98	0·93, 1·05	0·877
Soups	1·02	1·00, 1·05	1·03	0·98, 1·08	1·02	0·99, 1·05	0·817
Industrial baby foods	0·96	0·91, 1·02	0·94	0·88, 1·02	0·98	0·91, 1·07	0·632

* Includes three guardians other than parents.

† Includes early educators and early childhood education students.

‡ Independent-samples *t* test.

### Visual inspection of the validity

The Bland–Altman plots were made for eight food groups: (1) fresh berries and fruits, (2) fresh vegetables, (3) salads and grated vegetables, (4) warm vegetables, (5) desserts and sweet dairy products, (6) confectioneries and snacks, (7) soups and (8) porridges. The Bland–Altman plots showed different estimation errors (Supplementary Appendix Figs a–h). For fresh berries and fruits and soups, the 95 % limits of agreement were wide, indicating large between-person variation (Supplementary Appendix Figs a and g). Fresh vegetables had narrower 95 % limits of agreement but Bland–Altman plots showed that the larger the portion, the more biased estimation towards underestimation (Supplementary Appendix Fig. b). In the case of desserts and sweet dairy products, smaller portions tended to be more often overestimated whereas larger portions underestimated (Supplementary Appendix Fig. e). The same phenomenon was not seen with confectioneries and snacks (Supplementary Appendix Fig. f). Soups had the widest limits of agreement, and small portion sizes were underestimated while larger ones were overestimated (Supplementary Appendix Fig. g).

## Discussion

In this study, we assessed the validity of the age-specific food picture book in general and among parents and early educators. The proportion of correct estimations was high – 75 % of all estimations. It ranged from 100 % (fish fingers, Karelian pastries) to 36 % (cottage cheese) for individual food items. Statistically significant differences between parents and early educators were found in estimations of warm vegetables, berries, fresh fruits and pastries but the difference was small and geometric mean near 1 in all cases, so the differences have no relevance in practice.

Validation studies are used to quantify the level of precision of evaluation of a food amount for a variety of foods as well as to identify foods that cannot be evaluated accurately. The studies also increase the understanding of what extent food photographs improve estimates of nutrient intake^(^^18^^)^.

In our study, all food pictures and all portion sizes in every picture series were evaluated by 180 participants. We used the visual perception method^(^^18^^)^, which meant participants made a direct comparison between the food shown on the plate and the food in the photograph. It required the ability to think of the dimension and scaling of the photograph, shapes and density of food, food placement on the plate compared with the placement in the photograph, the dishes used and the proportions.

In the DAGIS study, the food picture book was intended to be used with a 3-d food record. Therefore, a real-time estimation was made based on the perception of foods in the photographs compared with the food shown on the dish. Study participants were recruited from a similar context to the DAGIS study where food diaries of the children were intended to be filled in by parents and early educators. To collect an accurate picture of the whole diet of young children, we often need to combine the food records of multiple adults.

In our study, there were significant differences in background characteristics (age, sex and education level) between parents and early educators. There were more male parent participants than male early educators. Parents were on average older and more educated than early educators. Nelson *et al*.^(^^20^^)^ proposed sex and age as potential confounding factors, but later studies^(^^1^^,^^2^^,^^21^^)^ came to the opposite conclusion. In a recent study^(^^13^^)^, educational level was not found to be associated with the accuracy of estimations. In a previous study in the Finnish context education level and BMI were not associated with differences in portion size estimation^(^^2^^)^. In studies about the food consumption of preschool-age children, the background of parents and early educators are likely to differ by sex, age and educational level also in real life. In our study, the group of early educators also included students, which decreased the mean age of this group. The personnel of day-care in Finland are dominated by women. Only 3 % of the personnel are men^(^^22^^)^. In that sense, our results reflect the reality.

According to power calculations, we aimed to have fifty-four estimates per food item. We had ten food items with fifty-seven evaluations and eighteen with fifty-three estimations, so in total, 29 % of the food items had enough estimations or almost enough to reach the aimed power. This was a limitation in this study. We tried to obtain 120 early educators and as many parents but recruiting parents was especially challenging. Evaluation situations were arranged at the time when parents were picking up their children from day-care, and they were often too busy to take part in the evaluation session. We included in the study only those preschools that had a kitchen close to the dining room and space enough to organise the evaluation situation, which restricted the number of preschools invited to the study.

Our study showed the accuracy of estimations ‘in the best case’, i.e. the food on the plates had the same appearance as in the photograph including the use of the same recipes and placement of foods on the plate. Portion sizes have been shown to be estimated more accurately when the portions on the plate have had exactly the same appearances as in the photograph^(^^5^^)^. When the food item served on the plate appeared exactly the same way as it appeared in the booklet, the exactly correct photograph was chosen 82 % of the time. Those portions that differed from the photographs were estimated correctly in 48 % of comparisons^(^^5^^)^. In our study preparation, weighing and plating in photographing and evaluation sessions were made by the same three people. Food portion sizes were exactly the same as in the food picture book. There was no option to choose a portion size between the ones shown in the food picture book, which is actually not the case in real life. In real-life situations, people dish up their own realistic portions, which differ from those shown in the food photographs. Here, the use of pre-weighed portions in an evaluation situation is a clear limitation in this study. The plates, cups, glasses and bowls were different than those in the food photograph book because that is the case in the data collection within the DAGIS study and because so many of the same dishes as we had in the food picture book were not available. Dishes are different in every child's home and in different preschools.

Of the food groups, porridges and soups were the least correctly estimated in our study. The proportion of correct estimations was 63 % for porridges and 60 % for soups. They were more often overestimated than underestimated. Soups and porridges were presented in bowls and deep plates whose dimensions and deepness may have been challenging to estimate. In the same kind of setting, Trolle *et al*.^(^^4^^)^ found the proportion of correct estimations for porridge to be 35 % and Naska *et al*.^(^^13^^)^ found the proportion for soups and porridges to be 66–72 %. In the Lillegaard *et al*.^(^^5^^)^ study, it was found that the proportion of correct estimation for porridge varies from 29 to 85 %, depending on the portion size. Small portion sizes were often overestimated. Our results are in line with these observations.

For vegetables and fruits, we noticed that some of the photographs were ambiguous. For cucumbers, sweet bell peppers, tomatoes, raw carrots, pineapples, water melon and kiwi fruit the same amount of vegetables or fruits were presented in photographs on the same plate but were chopped differently (e.g. slices, pieces; see Fig. 1). Our aim was to make estimation of the amount easier by presenting the same food amount in different shapes, but instead it made the estimation more problematic. With larger portions, the participants were confused if they needed to think of all the different vegetable shapes presented on the plate together or only the part of the plate where the vegetables were presented in the same shape as they had on the plate in front of them. This may be a reason why smaller portion sizes were more often correctly estimated and larger portions underestimated in the case of these particular vegetables and fruits. It was stated on the picture that the weights of the different shapes were the same. In the study by Trolle *et al*.^(^^4^^)^, the proportion of correct estimations for mixed salads was 28 % and 48 % for grated carrots. In our study, however, 61 % of estimations of salads and grated vegetables were correct.

In general, in previous studies with rather similar settings^(^^2^^,^^4^^,^^5^^)^ the total proportion of correct estimations has been about 50–60 % whereas in our study it was 75 %. It needs to be noted that in our study there was no option to choose a portion size between the ones shown in the food picture book, which was the case in the other studies mentioned. Also, in the other studies, only some of the portion sizes were the same as depicted in the food photographs^(^^2^^,^^4^^,^^5^^)^; however, in our study, food portion sizes were exactly the same.

Differences in the accuracy of portion size estimations between parents and early educators were small. Despite some significant differences in estimations of warm vegetables, berries, fresh fruits and pastries, the under- and overestimations were so small that they do not have relevance in practice. In addition, the percentages of correct estimations for pastries (90 %; *n* 103), warm vegetables (75 %; *n* 131) and berries and fruits (71 %; *n* 338) were high.

### Conclusions

In the present study, parents and early educators evaluated portion sizes with rather similar accuracy. The accuracy of estimations in general was high. However, pictures where vegetables and fruits were presented on the same plate in different shapes were confusing and often underestimated for larger portion sizes. These results suggest that the children's food picture book is a useful aid for both parents and early educators in the estimation of food portion sizes when the perception method is used.

## Supplementary material

For supplementary material accompanying this paper visit https://doi.org/10.1017/jns.2018.26. click here to view supplementary material

## References

[1] Turconi G, Guarcello M, Berzolari FG, (2005) An evaluation of a colour food photography atlas as a tool for quantifying food portion size in epidemiological dietary surveys. Eur J Clin Nutr 59, 923–931. 15928683 10.1038/sj.ejcn.1602162

[2] Ovaskainen M-L, Paturi M, Reinivuo H, (2008) Accuracy in the estimation of food servings against the portions in food photographs. Eur J Clin Nutr 62, 674–681. 17440523 10.1038/sj.ejcn.1602758

[3] Korkalo L, Erkkola M, Fidalgo L, (2013) Food photographs in portion size estimation among adolescent Mozambican girls. Public Health Nutr 16, 1558–1564. 22874096 10.1017/S1368980012003655 PMC10271437

[4] Trolle E, Vandevijvere S, Ruprich J, (2013) Validation of a food quantification picture book targeting children of 0–10 years of age for pan-European and national dietary surveys. Br J Nutr 110, 2298–2308. 23803561 10.1017/S0007114513001694

[5] Lillegaard ITL, Overby NC & Andersen LF (2005) Can children and adolescents use photographs of food to estimate portion sizes? Eur J Clin Nutr 59, 611–617. 15702127 10.1038/sj.ejcn.1602119

[6] Foster E, Matthews JNS, Lloyd J, (2008) Children's estimates of food portion size: the development and evaluation of three portion size assessment tools for use with children. Br J Nutr 99, 175–184. 17697426 10.1017/S000711450779390X

[7] Foster E, Adamson AJ, Anderson AS, (2009) Estimation of portion size in children's dietary assessment: lessons learnt. Eur J Clin Nutr 63, Suppl. 1, S45–S49. 19190643 10.1038/ejcn.2008.64

[8] Foster E, Hawkins A & Adamson AJ (2010) Young Person's Food Atlas – Pre-School. London: Food Standards Agency.

[9] Andersen LF, Lioret S, Brants H, (2011) Recommendations for a trans-European dietary assessment method in children between 4 and 14 years. Eur J Clin Nutr 65, Suppl. 1, S58–S64. 21731007 10.1038/ejcn.2011.88

[10] Foster E & Adamson A (2014) Challenges involved in measuring intake in early life: focus on methods. Proc Nutr Soc 73, 201–209. 24555806 10.1017/S0029665114000020

[11] Subar AF, Crafts J, Zimmerman TP, (2010) Assessment of the accuracy of portion size reports using computer based food photographs aids in the development of an automated self-administrative 24-hour recall. J Am Diet Assoc 110, 55–64. 20102828 10.1016/j.jada.2009.10.007 PMC3773715

[12] Foster E, Hawkins A, Simpson E, (2014) Developing an interactive portion size assessment system (IPSAS) for use with children. J Hum Nutr Diet 27, Suppl. 1, 18–25. 23682796 10.1111/jhn.12127

[13] Naska A, Valanou E, Peppa E, (2016) Evaluation of a digital food photography atlas used as portion size measurement aid in dietary surveys in Greece. Public Health Nutr 19, 2369–2376. 26917048 10.1017/S1368980016000227 PMC10271068

[14] de Boer EJ, Slimani N, van ‘t Veer P, (2011) The European Food Consumption Validation Project: conclusions and recommendations. Eur J Clin Nutr 65, Suppl. 1, S102–S107. 21731001 10.1038/ejcn.2011.94

[15] Foster E, Matthews JNS, Nelson M, (2006) Accuracy of estimates of food portion size using food photographs – the importance of using age-appropriate tools. Public Health Nutr 9, 509–514. 16870024 10.1079/phn2005872

[16] Määttä S, Lehto R, Nislin M, (2015) Increased health and well-being in preschools (DAGIS): rationale and design for a randomized controlled trial. BMC Public Health 15, 402. 25927298 10.1186/s12889-015-1744-z PMC4419478

[17] Erkkola M, Saloheimo T, Hauta-alus H, (2016) Burden of allergy diets in Finnish day care reduced by change in practices. Allergy 71, 1453–1460. 27117067 10.1111/all.12902

[18] Nelson M & Haraldsdóttir J (1998) Food photographs: practical guidelines I. Design and analysis of studies to validate portion size estimates. Public Health Nutr 1, 219–230. 10933422 10.1079/phn19980038

[19] Noether GE (1987) Sample size determination for some common nonparametric tests. J Am Stat Assoc 82, 645–647.

[20] Nelson M, Atkinson M & Darbyshire S (1996) Food photography II: use of food photographs for estimating portion size and the nutrient content of the meals. Br J Nutr 76, 31–49. 8774215 10.1079/bjn19960007

[21] De Keyzer W, Huybrechts I, De Maeyer M, (2011) Food photographs in nutritional surveillance: errors in portion size estimation using drawings of bread and photographs of margarine and beverages consumption. Br J Nutr 105, 1073–1083. 21092383 10.1017/S0007114510004551

[22] Statistics Finland (2008) Liitetaulukko 1. Suurimmat naisten ammattiryhmät (naisia 90–100 % ammattiryhmästä) vuonna 2008 (Appendix table 1. The largest professional women's groups (women 90–100 % of the professional category) in 2008). https://www.stat.fi/til/tyokay/2008/04/tyokay_2008_04_2010-12-03_tau_001_fi.html (accessed December 2018).

