# Supplementary material for: Accuracy in the estimation of children's food portion sizes against a food picture book by parents and early educators
Source: J Nutr Sci. 2018 Dec 27;7:e35. doi: 10.1017/jns.2018.26 (PMC6313402; doi:10.1017/jns.2018.26)
Supplement: Supplementary file 1 [file S2048679018000265sup001.pdf]

Appendix. Bland-Altman plots showing the mean difference (____) and 95 % limits of agreement (- - -) between estimated and actual portion sizes for a) fresh berries and fruits (n 338), b) fresh vegetables (n 285), c) salads and grated vegetables (n 114), d) warm vegetables (n 131), e) desserts and sweet dairy (n 248), f) confectionaries and snacks (n 248), g) soups (n 212) and h) porridges (n 64).

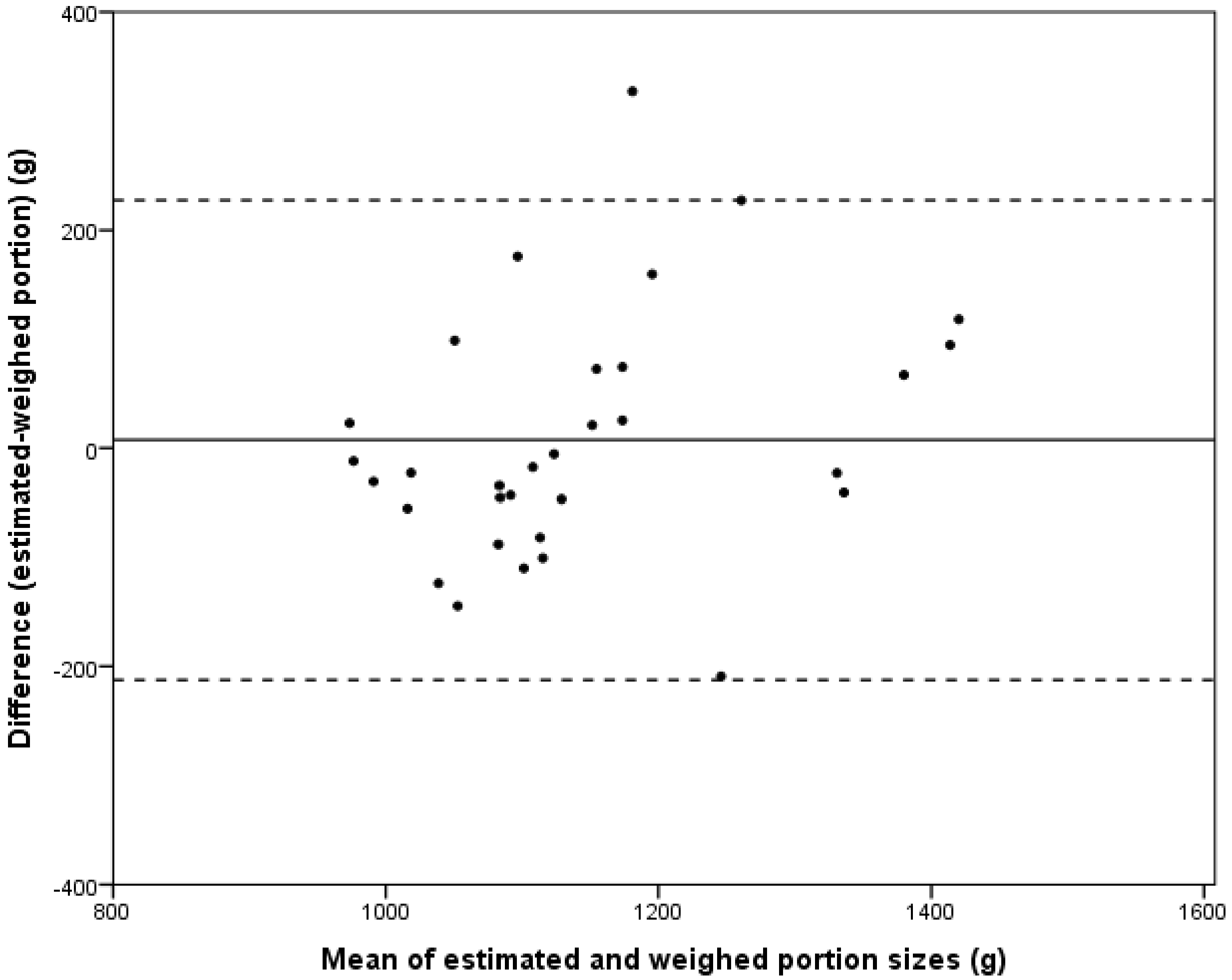

a) Fresh berries and fruits (*n* 338)

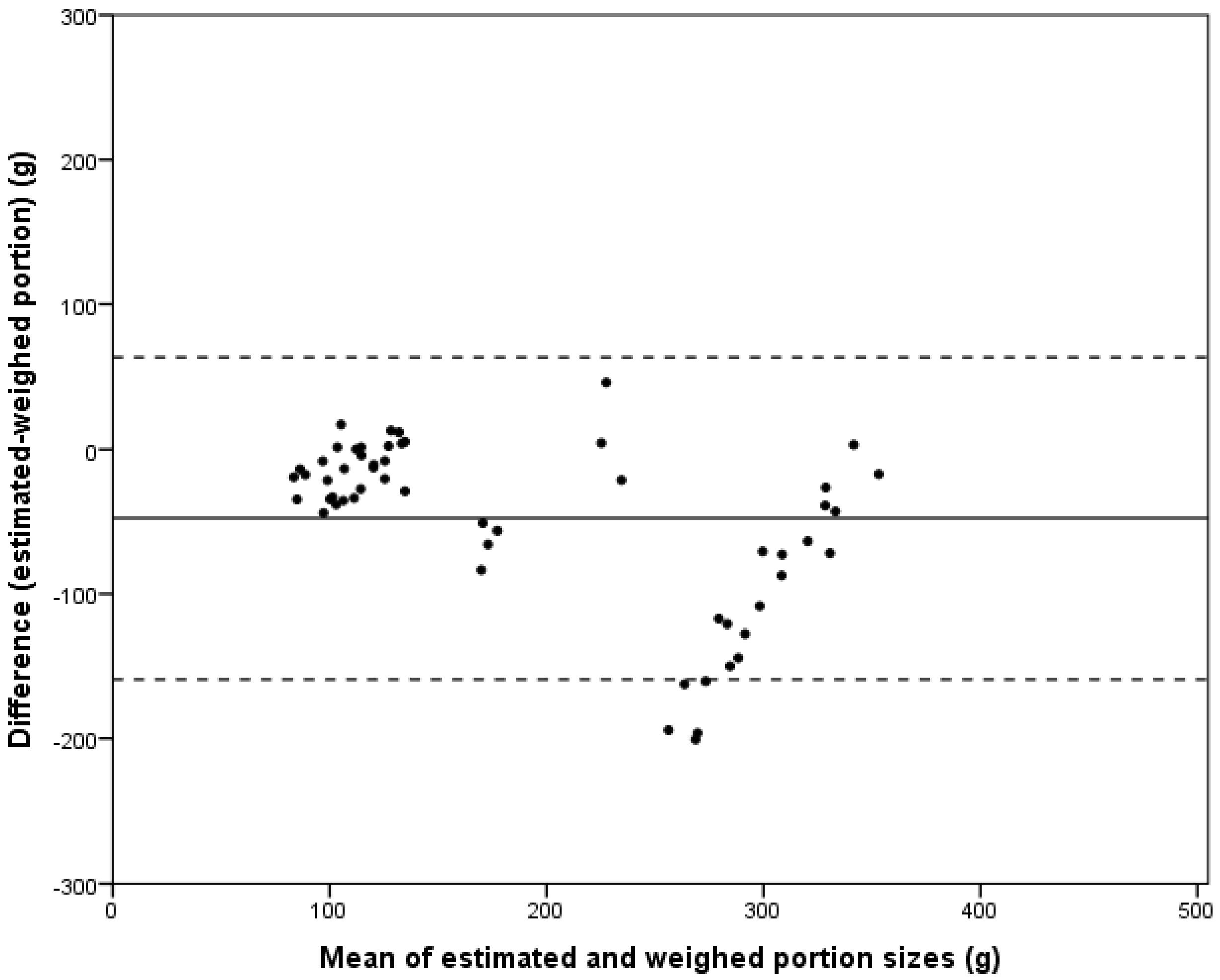

b) Fresh vegetables (*n* 285)

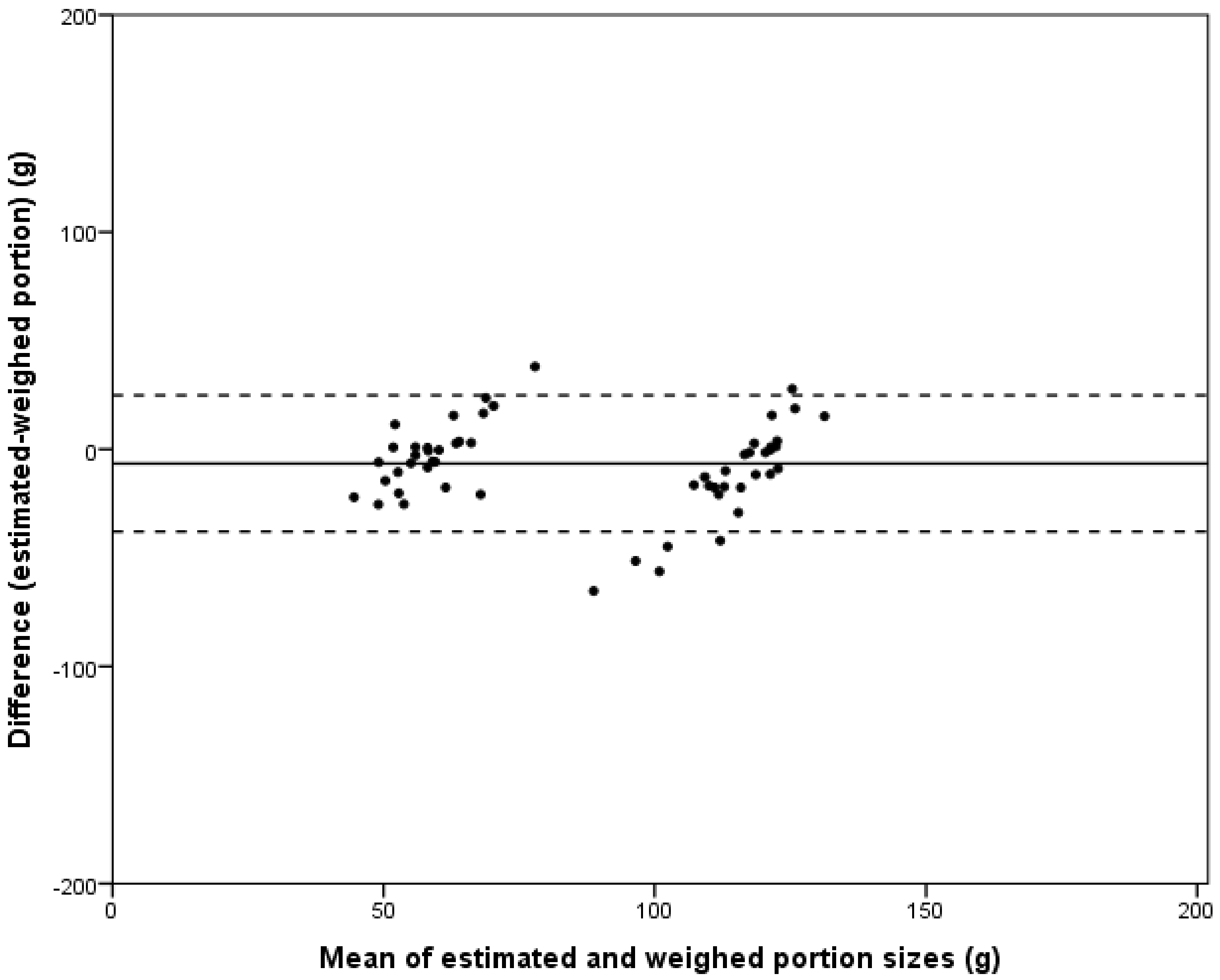

c) Salads and grated vegetables (*n* 114)

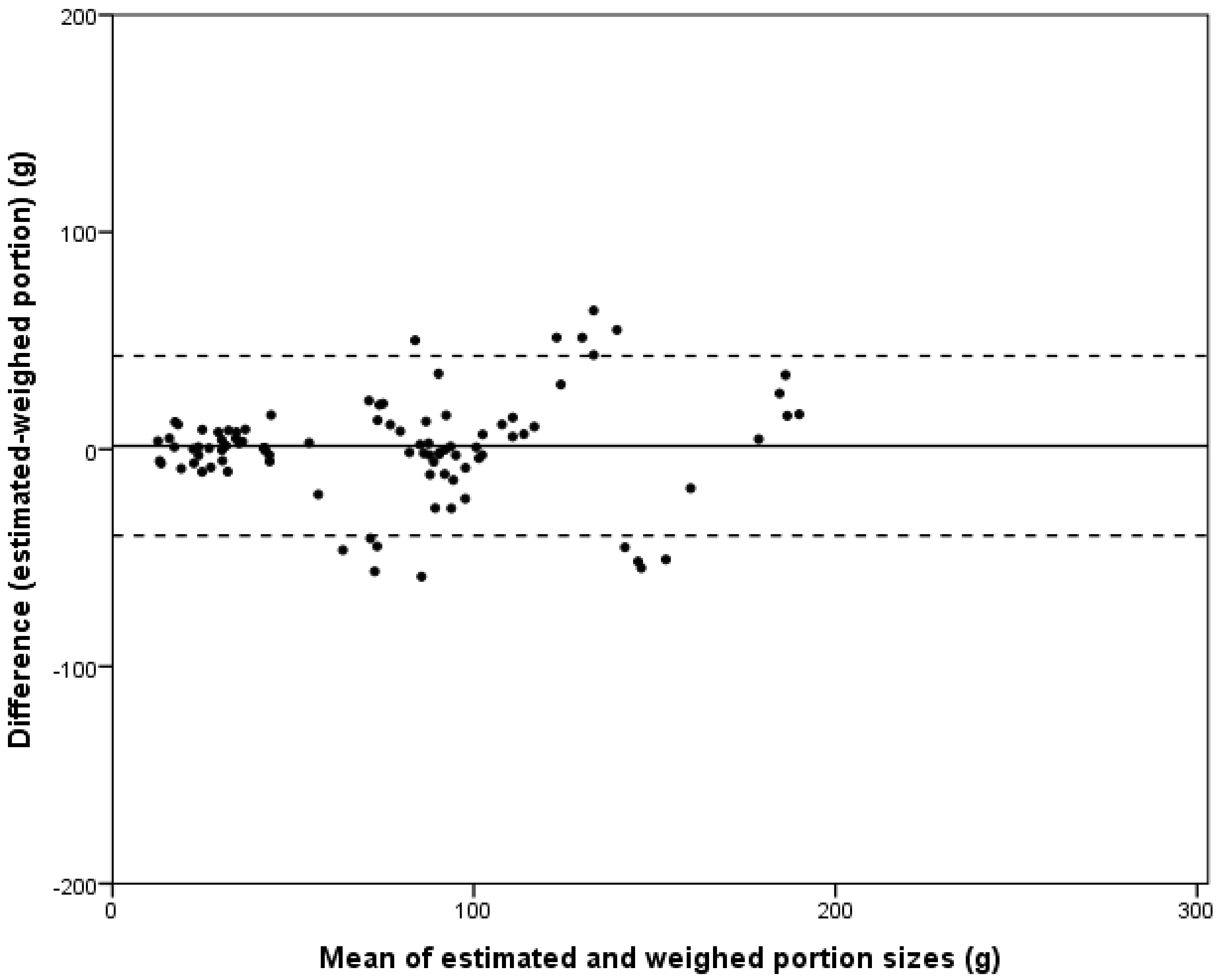

d) Warm vegetables (*n* 131)

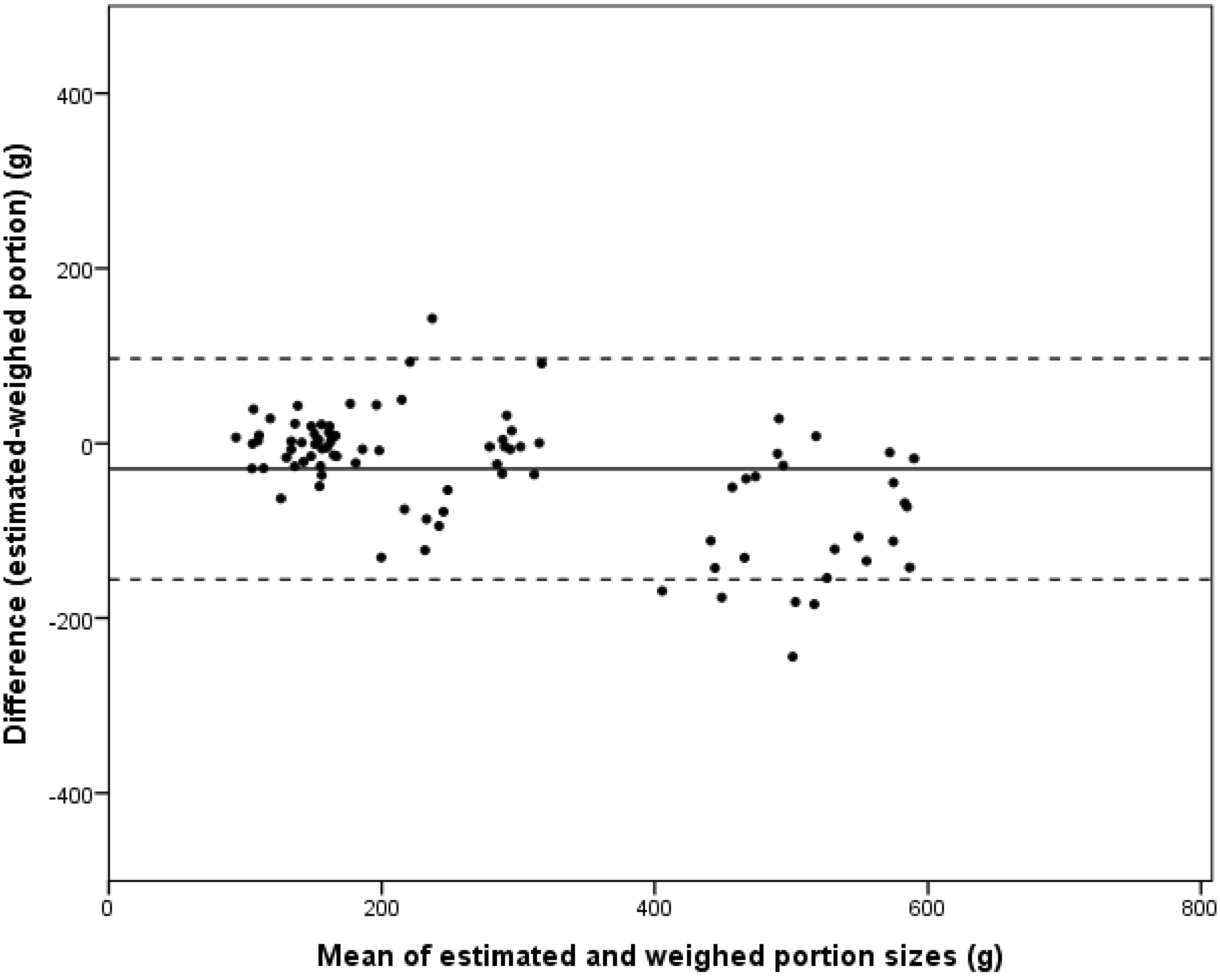

e) Desserts and sweet dairy (*n* 248)

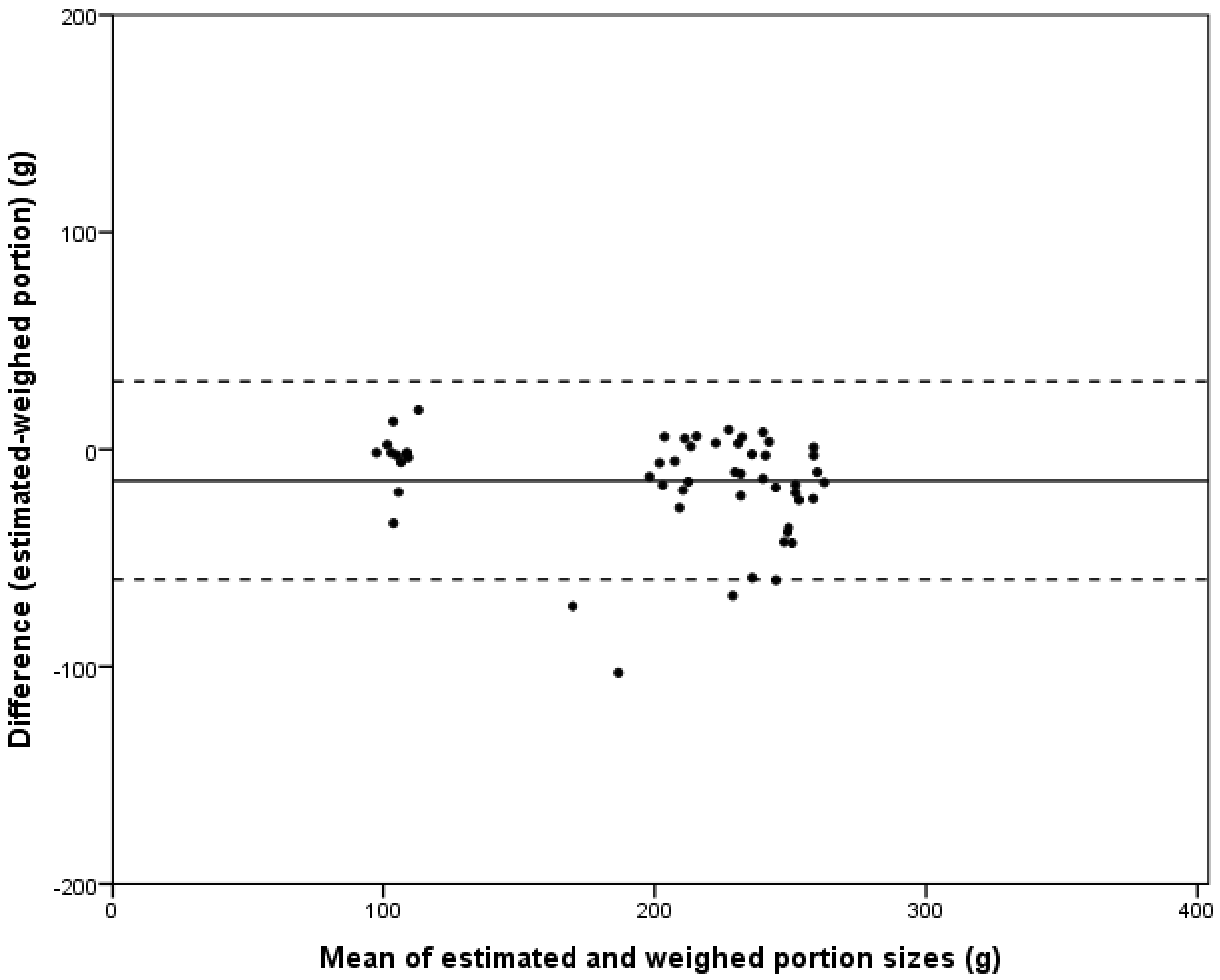

f) Confectionaries and snacks (*n* 248)

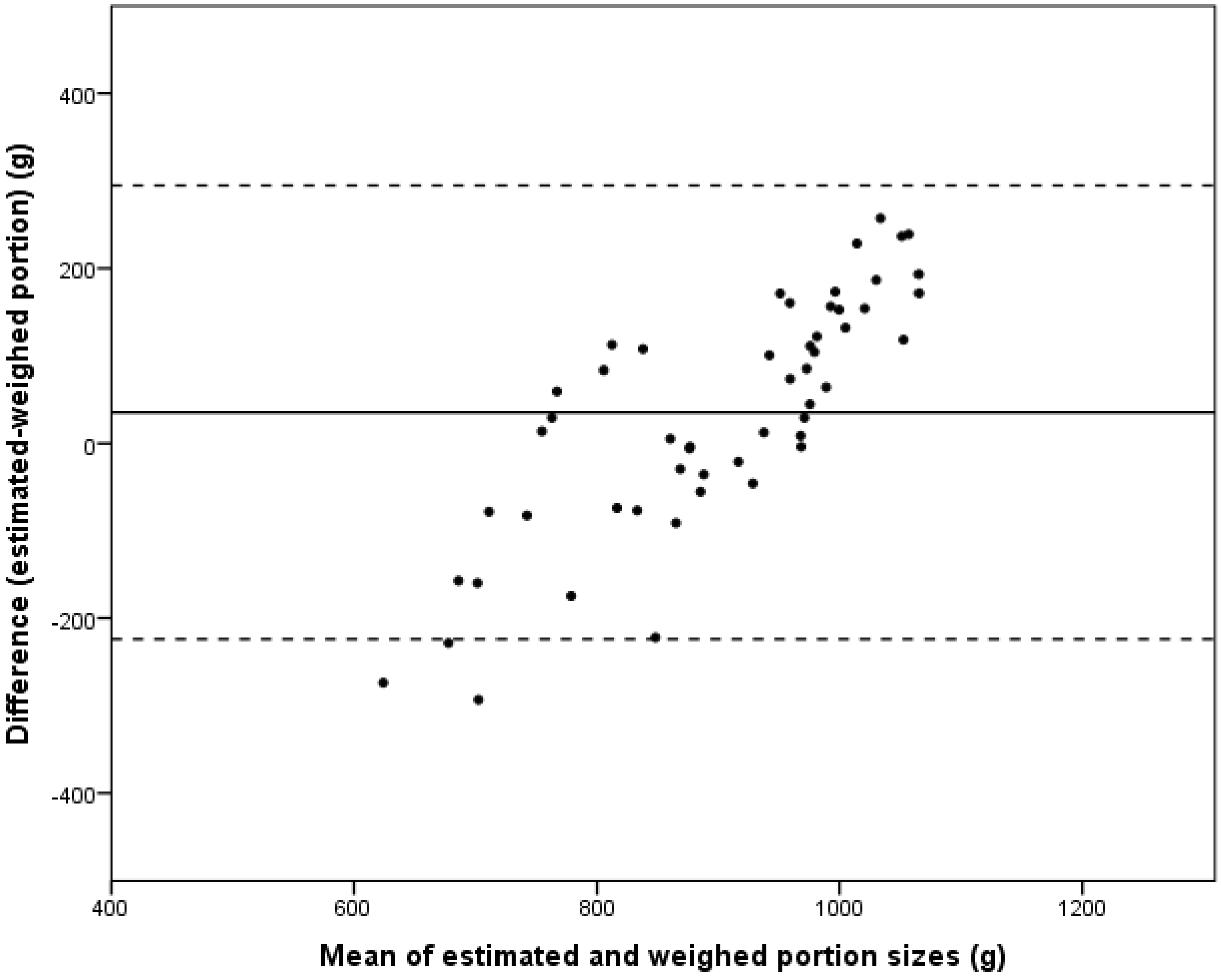

400
200
0
-200
-400
Difference (estimated-weighed portion) (g)
400
600
800
1000
1200
Mean of estimated and weighed portion sizes (g)

g) Soups (*n* 212)

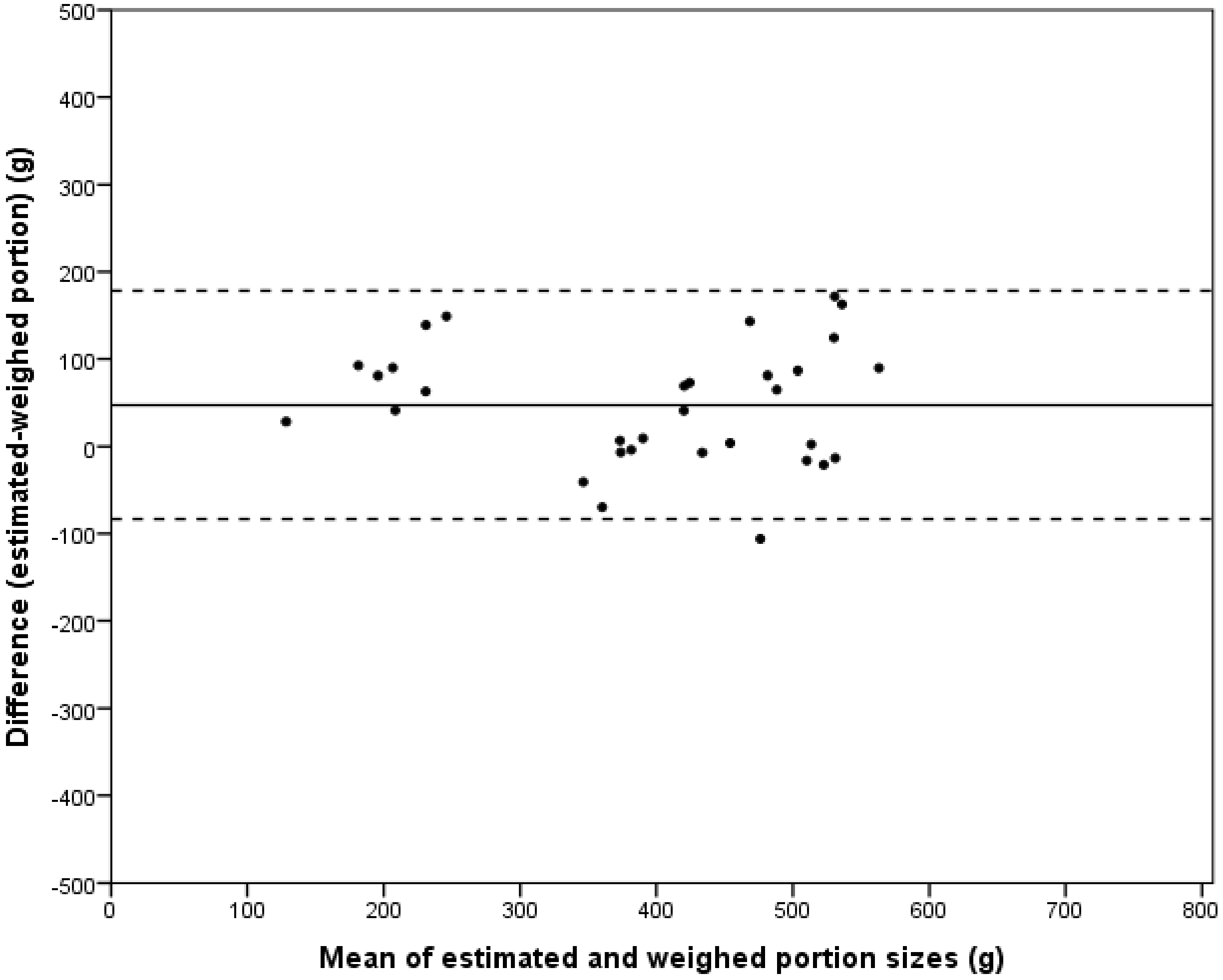

h) Porridges (*n* 64)
